# Optimizing blueberry (*Vaccinium corymbosum* L.) yield with strategic foliar application of putrescin and spermidine at key growth stages through biochemical and anatomical changes

**DOI:** 10.3389/fpls.2025.1564026

**Published:** 2025-05-30

**Authors:** Amir Ali Karami, Vahid Abdossi, Marzieh Ghanbari Jahromi, Abdolhosain Aboutalebi Jahromi

**Affiliations:** ^1^ Department of Horticultural Science and Agronomy, Science and Research Branch, Islamic Azad University, Tehran, Iran; ^2^ Department of Horticultural Science, Jahrom Branch, Islamic Azad University, Jahrom, Iran

**Keywords:** antioxidant potential, fruit yield, polyamines, spraying schedule, stomatal density

## Abstract

Foliar spraying of polyamines, such as putrescine (Put) and spermidine (Spd), has been shown to improve plant and fruit yield. However, the optimal time for spraying these polyamines is still uncertain. The present study was conducted to evaluate plant growth, stomatal parameters, and biochemical attributes of blueberry (*Vaccinium corymbosum* L.) under Put and Spd application at various spraying times. Plants received spray treatments with doses of 0.5 and 1 mM of Put and Spd during bloom, fruit development, and ripening stages. According to the findings, both Put and Spd enhanced fruit and plant yield, although Put had a greater effect. The best timing to apply foliar spraying was observed during the fruit development stage. In plants sprayed at the fruit development stage, Put at 1 mM led to increased plant height (11%), SPAD value (17%), fruit yield (80%), fruit firmness (34%), anthocyanin (31%), total phenolic content (TPC, 14%), total flavonoid content (TFC, 35%), stomatal length (45%), stomatal width (40%), nitrogen (N, 43%), phosphorous (P, 21%), and potassium (K, 18%), but decreased antioxidant capacity (IC50, 36%) and stomatal density (19%) relative to the control. In response to the management for bloom, fruit development, and ripening stages, fruit yield in plants sprayed with Put at 1 mM increased by 55, 80, and 64%, respectively. Heat map analysis revealed the maximum variability among traits associated with fruit yield under the treatments. The research suggests that strategic foliar application of Put and Spd at specific growth stages can enhance blueberry yield and quality, with the fruit development stage showing promising results.

## Introduction

Blueberry (*Vaccinium corymbosum* L.) is a perennial flowering plant from the family Ericaceae and is known for its nutritious fruits. It produces white or pink flowers and blue-black berries when ripe. Blueberries are an important fruit crop valued for their health benefits, flavor, and culinary versatility (Krishna et al., 2023). To meet the growing demand for high-quality blueberries, it is essential for growers to implement effective cultivation practices that optimize both yield and fruit quality (Redpath et al., 2021). One such practice that has gained attention in recent years is the strategic foliar application of polyamines (Tyagi et al., 2023).

Putrescine (Put) and spermidine (Spd) are essential polyamines in plants, regulating cell division, differentiation, and expansion. Spd promotes cell proliferation and differentiation, while Put is crucial for cell elongation and tissue development (Zhong et al., 2023; Huang et al., 2024). These polyamines also help plants adapt to environmental stresses by regulating gene expression, scavenging reactive oxygen species and maintaining cellular homeostasis. Exogenous application of Put and Spd can positively impact plant growth and stress responses, potentially enhancing crop health and productivity (Mahdavian et al., 2021; Liu et al., 2023; Prajapati et al., 2024). The foliar application of Put and Spd approach can meet the growing demand for high-quality blueberries while ensuring sustainable crop production practices (Huang et al., 2024).

The use of Spd and Put in blueberry cultivation is particularly beneficial during key growth stages. During these stages, the demand for nutrients is high as the plants undergo rapid growth and development to produce high-quality fruit. Blueberry growth stages include dormant stage (winter), bud swell, flowering (blooming), fruit development, and harvest. Monitoring these stages is crucial for activities like pruning, fertilization, pest control, and irrigation, leading to optimal yield and fruit quality (Nabetani et al., 2017). Foliar application of Spd and Put at these critical times can help support healthy fruit development, enhance fruit quality traits such as size, color, and taste, and ultimately enhance market competitiveness for blueberry growers (Jangra et al., 2023).

According to reports, the beneficial use of Put can improve fruit taste and quality (Hanif et al., 2020; Piñero et al., 2021; Khodabakhshi et al., 2023; Prajapati et al., 2024). Furthermore, studies on the beneficial effects of Spd on certain fruit quality traits have been documented (Hadjipieri et al., 2021; Huang et al., 2024). The impact of these polyamines on blueberry growth and fruit quality is still unclear. The goal of this study was to investigate the possible advantages and results of maximizing blueberry production and quality by strategically applying Spd and Put during the optimal timing of foliar application throughout the growth period. Fruit quality was evaluated using biochemical parameters such as anthocyanin, phenolic content, flavonoids, and antioxidant capacity. Plant growth and development were also assessed through measurements of photosynthetic rate, stomatal traits, and leaf mineral content.

Fruit quality was examined using biochemical parameters such as anthocyanin, phenol, flavonoids, and antioxidant capacity. Plant growth and development were examined through photosynthetic rate, stomatal changes, and leaf minerals. The study investigated the effects of Spd and Put on fruit development, plant growth, and crop yield, providing valuable insights for blueberry producers to enhance their production methods, optimize output potential, and achieve long-term success in blueberry farming.

## Materials and methods

### Experimental treatments and growth circumstances

The information provided describes a pot experiment conducted in 2023 using a factorial experiment based on a completely randomized design (CRD) with three replicates for each treatment. Two-year-old blueberry (*Vaccinium corymbosum* L.) cv. Brijita plants were obtained by the Biotechnology Institute in Rasht, Iran. The plants were cultivated in 3-liter pots in an open area with an average relative humidity of 80% and an average temperature of 17°C during the growth period.

The phenological stages were determined according to the BBCH scale. Treatments were applied at BBCH 60 (blooming), BBCH 71 (fruit development), and BBCH 80 (fruit ripening) (Wichura et al., 2024). During each of these stages—bloom, fruit development, and ripening—Put and Spd were applied at five concentrations: control (0 mM), Put at 0.5 and 1 mM, and Spd at 0.5 and 1 mM. Foliar Applications were made at the onset of each stage. The growth medium consisted of peat moss, coco peat, and perlite in a 70:20:10 ratio. Plants were nourished weekly with 500 mL of Hoagland solution per pot, applied as irrigation water. Electrical conductivity (EC) was maintained at 1.5 dS m^-1^, and pH was adjusted to 5.7–5.9 to optimize nutrient uptake. Irrigation scheduling was based on soil field capacity, with each plant receiving 500 mL of water every three days.

Fruit and leaf samples were collected from two-year-old blueberry plants during the fruit ripening stage, approximately 95 days after the start of the experiment. Sampling was conducted when the majority of fruits had reached full maturity based on visual assessment of color, size, and firmness. To ensure reliable and representative data, a randomized sampling method was used. From each treatment group, three pots (or plants) were randomly selected. From each selected plant, fully ripened fruits and fully expanded, healthy leaves from the mid-canopy were picked. All samples were collected in the early morning to minimize variation due to diurnal changes. Fruits were immediately weighed and then freeze-dried until further biochemical assessments were performed.

For anatomical analysis and leaf mineral content determination, the leaves were dried in the shade.

### Plant height

At the end of the experiment, the plant heights were measured from the base of the stem to the tip of the branch using a ruler.

### Chlorophyll index measurement (SPAD value)

SPAD values of blueberry leaves were measured using a Field Scout Chlorophyll Meter to assess relative chlorophyll content. The experiment involved tagging four randomly selected terminal and lateral branches on each plant. Branch growth was measured in April, May, and June using a spectral reflectance method measuring light at 700 and 840 nm from both ambient and reflected sources (Monostori et al., 2016).

### Fruit yield and fruit firmness

Fruit yield was calculated by measuring the weight of all fruits per plant. The firmness of fruits was assessed using a penetrometer (FT011 Fruit Firmness Tester; Wagner Instruments, Italy). Measurements were taken by inserting the penetrometer into two opposite sides of each fruit (Yaghubi et al., 2019).

### Preparation of fruit extraction

Fruit tissue samples were ground with liquid nitrogen. Subsequently, 5 g of fruit tissue was homogenized in 10 mL of 50 mmol L^–1^ phosphate buffer (pH 7.8). To determine additional analyses, the homogenate was centrifuged at 15000×*g* for 20 min at 4°C. The supernatant, which is fruit extract, was collected (Yaghubi et al., 2019).

### Anthocyanins measurement

Two buffer systems—25 mM KCl buffer (pH 1.0) and 0.4 M Na acetate buffer (pH 4.5)—were used to quantify total anthocyanin. KCl buffer was used to dilute the samples until A510 was within the spectrophotometer’s linear range. Following that, the sample was diluted using the same dilution factor in Na acetate buffer. The absorbance was measured at 510 and 700 nm after the two buffers were incubated for 15 min. The total anthocyanin content was calculated using the method outlined by Giusti and Wrolstad (2001): Anthocyanin = [(A × MW × DF ×100)/MA] where ‘A’ stands for corrected absorbance (A510-A700), MW is the molecular weight of Cyan-3glu, MA is molar extinction coefficient. Anthocyanin content was measured as mg of Cyan-3-glu per 100 g^-1^ equivalents.

### Total phenolic content determination

The Folin–Ciocalteu colorimetric method was used to determine the total phenolic contents (TPC) in fruits, seeds, and bark extracts. A standard gallic acid (GA) solution was prepared by dissolving 10 mg in methanol. Various concentrations of GA solutions were prepared, with each concentration added to a final volume of 10 mL. The blue-colored mixture was shaken and incubated for 30 min at 40°C. The absorbance was measured at 760 nm against a blank, and the FCR reagent oxidized phenols in plant extracts, resulting in a dark blue color. The absorbance was then measured by a UV-visible spectrophotometer. Samples were prepared in triplicate for each analysis, and the average absorbance value was used to plot the calibration curve to determine the level of phenolics in the extracts. The total phenolic content was expressed as mg GA equivalents per gram of sample in dry weight (mg g^-1^). The total phenolic contents were calculated using the formula of C = (c × V)/m, where C = total phenolic content mg GA g^-1^ extract, c = concentration of GA obtained from the calibration curve in mg mL^-1^, V = volume of extract in mL, and m = mass of extract in g (Singleton, 1999).

### Total flavonoid content determination

The TFC in the extracts were determined using an aluminum chloride colorimetric assay. A stock solution of quercetin (QE) was prepared by dissolving 4 mg in 1 mL of methanol. This solution was diluted to create different concentrations of QE, which were then added to a test tube containing distilled water. The mixture was then mixed with 5% NaNO_2_, 10% AlCl_3_, and NaOH. The volume of the mixture was then increased to 10 mL. The extracts were prepared in the same manner, and the absorbance was measured using a spectrophotometer at 510 nm. The average absorbance value was used to calculate the total flavonoid content, which was expressed as mg QE g^-1^ using the linear equation based on the standard calibration curve (Shraim et al., 2021).

### Antioxidant capacity (IC50 value)

The study aimed to determine the *in vitro* antioxidant activities of extracts using the DPPH free radical scavenging assay, which is a quick and easy method to analyze the scavenging potential of antioxidants. The DPPH solution (0.1 mM) was prepared by dissolving 0.39 mg of DPPH in methanol and storing it at -20°C. A stock solution of different extracts at 1 mg mL^-1^ was prepared by dissolving the required quantity of each extract in methanol. From the sample stock solution, 25, 50, 75, and 100 μg mL^-1^ solutions of each extract were prepared. The antioxidant potential of the extracts was evaluated by adding 1 mL of DPPH solution to the sample solutions of different concentrations and incubating them at room temperature for 30 min in the dark. A control was prepared by mixing 1 mL of methanol and 1 mL of DPPH solution. The absorbance of the solutions was measured using a spectrophotometer at 517 nm, with ascorbic acid as the standard. The 50% inhibitory concentrations (IC50 values) of the extracts were calculated from graphs as concentration versus percentage inhibition, and the radical scavenging activity was expressed as the percentage of inhibition. IC50 values represented the concentration required to inhibit 50% of DPPH radicals (Nithianantham et al., 2011).

### Stomatal size and density

Using scanning electron microscopy (SEM; SU 3500, Hitachi, Japan), dried leaf samples were examined to determine the size and density of the stomata. In the vacuum coating facility (SG 110, Iran), dried samples were coated with gold for this purpose, in accordance with the methodology described by Khosropour et al. (2019). To measure the anatomical features, Image J software was used.

### Leaf minerals

The study aimed to determine the macronutrient content of leaves. Leaf samples, in the form of oven-dried powder, were digested using AR-grade concentrated sulfuric acid and hydrogen peroxide. The aliquot (peroxide-digested material) was used to estimate the percentage content of macronutrients on a dry weight basis. Leaf nitrogen (N) was measured with the Kjeldahl method by titration (Sáez-Plaza et al., 2013). At this stage, 0.1 N sulfuric acid was added dropwise to the ammonium borate solution using a buret. The volume of acid consumed (V) was recorded when the solution changed color from green to red. The percentage of N in the sample is calculated as follows:


N%=A*N*1.4/W

N = normality of acid, A = volume of acid consumed, and W = sample weight.

Phosphorous P content was estimated using a standard graph prepared by graded dilutions of monopotassium phosphate. Potassium (K) contents in the leaves were was determined using flame photometry (Cotton, 1945).

### Data analysis

After collecting the data, they were analyzed using the SAS (version 9.3, SAS Institute, Cary, NC, USA) program for factorial design. Data means were compared using Duncan’s multiple range test at a 5% probability level (*P ≤* 0.05). PCA was conducted by XLSTAT, and a heat map was developed by CIMMiner at https://discover.nci.nih.gov/cimminer/home.do.

## Results

### Plant height and SPAD value

**Figure 1:**
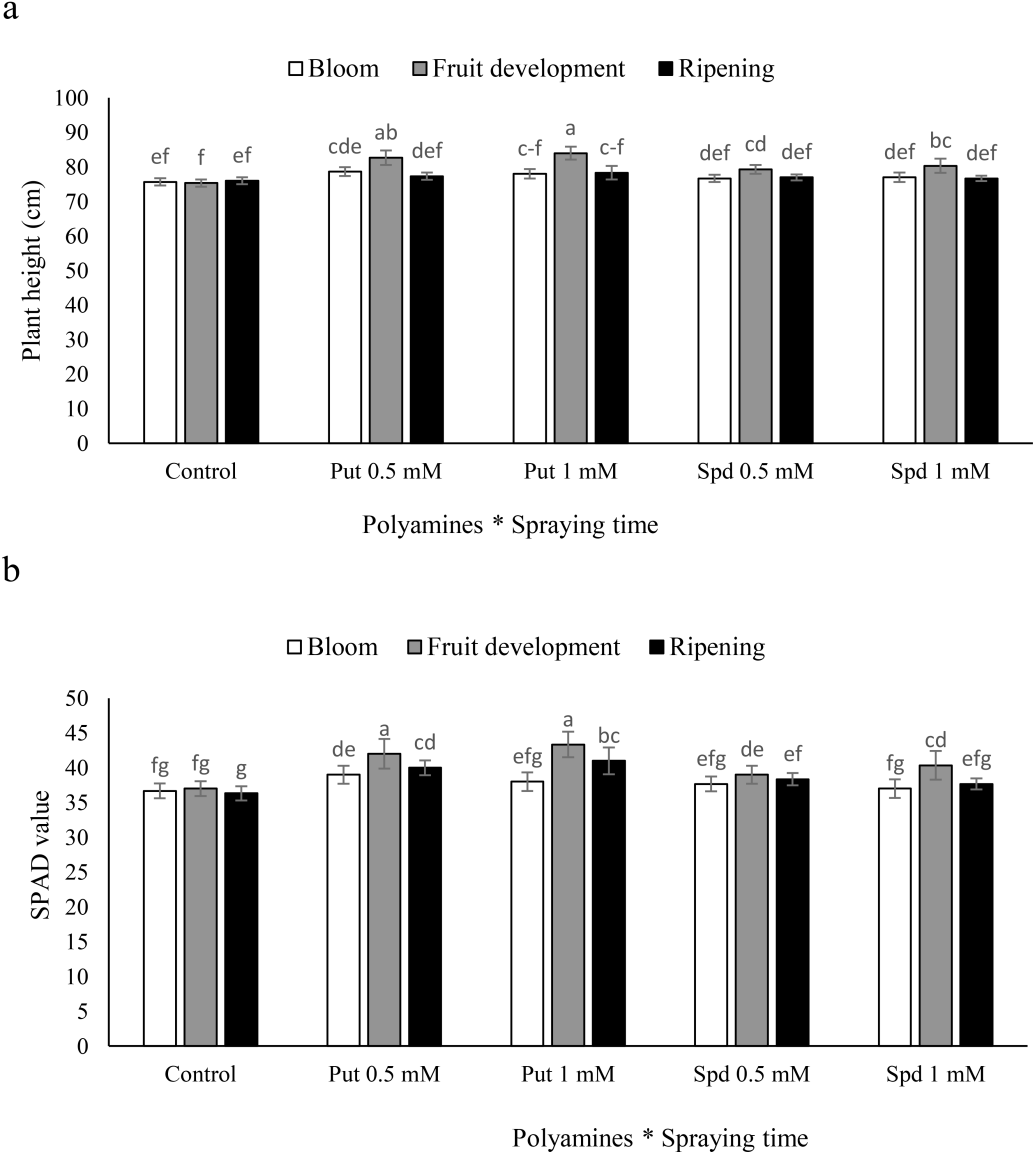
Plant height **(a)** and SPAD value **(b)** of blubbery plants under foliar applied putrescine (Put) and spermidine (Spd). Values are means ± standard error (SE) and different letters show 650 statistically significant differences among treatments at *P ≤* 0.05.

The study showed that foliar-applied Put and Spd had positive effects on plant growth. It indicated that 1 mM Put during fruit development could boost plant height from 75.3 to 84 cm, demonstrating the potential of plant growth regulators to enhance plant development. The study found that specific concentrations of Put and Spd were crucial for promoting plant growth, with 1 mM Put treatment having a more significant effect (**Figure 1a**). The fruit development stage was the most effective time for applying Put and Spd to blueberry plants to boost chlorophyll index. Specifically, when these polyamines were applied during the fruit development stage, the chlorophyll index of the plants increased significantly compared to the control. For example, Put at 0.5 and 1 mM led to 13% and 17% increases in the SPAD value, respectively. Similarly, the SPAD value increased by 6% and 10% at 0.5 and 1 mM of Spd, respectively. These findings suggest that both Put and Spd could effectively promote chlorophyll production when applied during the fruit development stage. In contrast, the blooming stage did not show significant changes in the SPAD value when polyamines were applied. Additionally, at the harvest stage, Put at 0.5 and 1 mM led to significant increases in the SPAD value by 11% and 14%, respectively. Spd at 0.5 and 1 mM concentrations resulted in smaller increases of 6% and 4% in the SPAD value, respectively (**Figure 1b**).

### Fruit yield and fruit firmness

**Figure 2:**
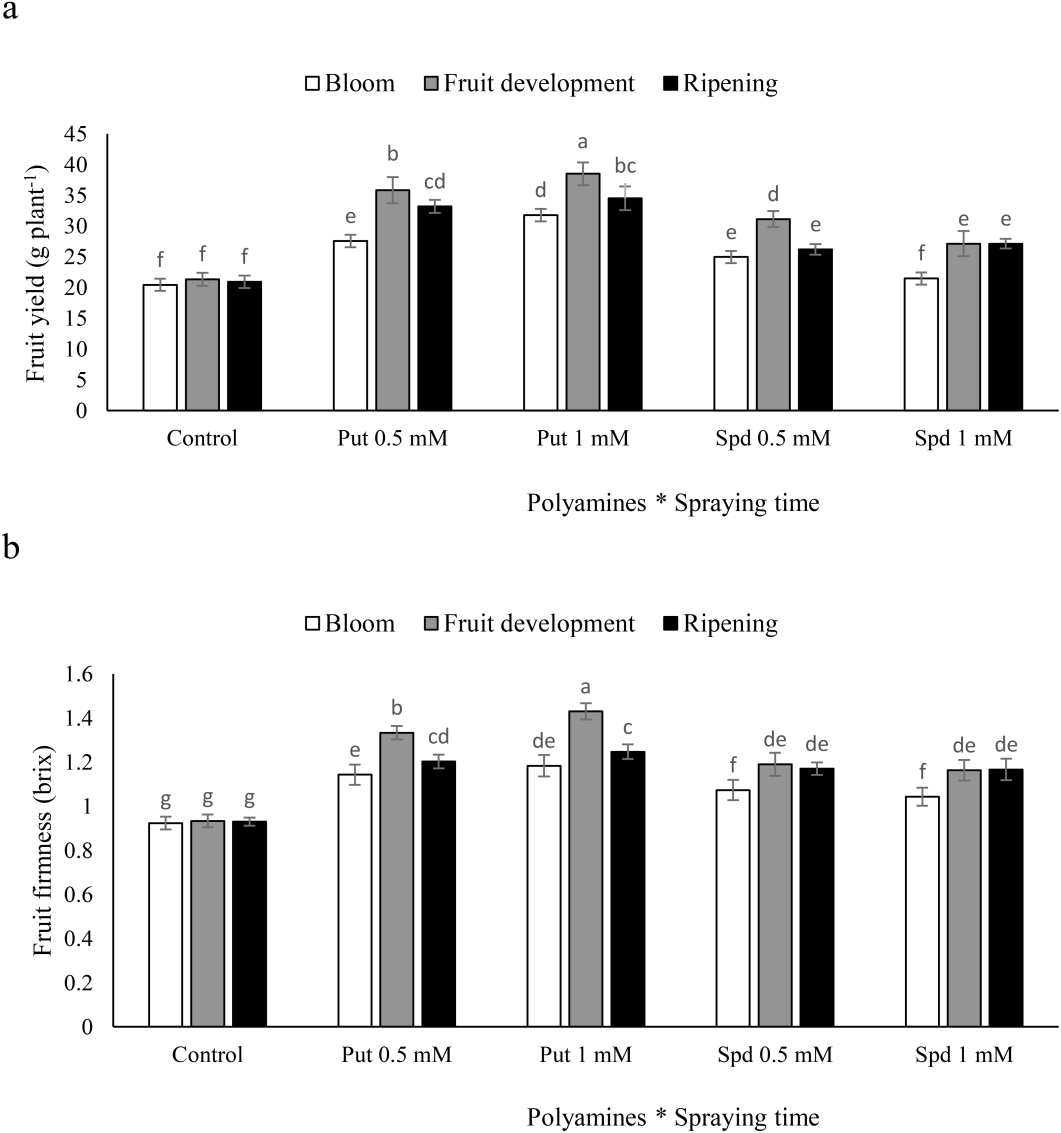
Fruit yield **(a)** and fruit firmness **(b)** of blubberers under foliar applied putrescine (Put) and spermidine (Spd). Values are means ± standard error (SE) and different letters show 668 statistically significant differences among treatments at *P ≤* 0.05.

The fruit development stage is the most effective time for Put and Spd on blueberry plants to maximize fruit yield. The application of Put during the fruit development stage led to a notable increase in fruit yield, with significant improvements observed at both 0.5 and 1 mM concentrations. Specifically, when plants were sprayed with Put at 0.5 and 1 mM during the fruit development stage, there were substantial increases in fruit yield by 65% and 70%, respectively. Spd at 0.5 and 1 mM also resulted in significant increases in fruit yield, with increments of 45 and 27%, respectively. These findings demonstrate the potential of polyamines to enhance fruit yield when applied during the fruit development stage. In contrast, the increases in fruit yield were smaller when polyamines were applied at the blooming and harvest stages. The most effective treatment was the application of Put at 1 mM concentration, which led to increases of 55% for the blooming stage, 80% for the fruit development stage, and 64% for the harvest stage compared to the control. This suggests that the fruit development stage may be the critical period for maximizing the effects of polyamines on fruit yield in blueberry plants (**Figure 2a**). The timing of polyamine application had varied effects on fruit firmness in blueberry plants. The application of Put resulted in significant increases in fruit firmness, with different responses observed depending on the stage at which the polyamines were sprayed. Specifically, when Put was sprayed at the harvest stage, there were notable improvements in fruit firmness. In comparison to the control, the application of Put at 1 mM was shown to be the most beneficial, resulting in significant improvements in fruit firmness of 28% during the blooming stage, 53% during the fruit development stage, and 34% during the harvest stage (**Figure 2b**). These findings highlight the importance of considering the timing and concentration of polyamine applications to optimize fruit firmness in blueberry plants.

### Fruit anthocyanin and fruit IC50

**Figure 3:**
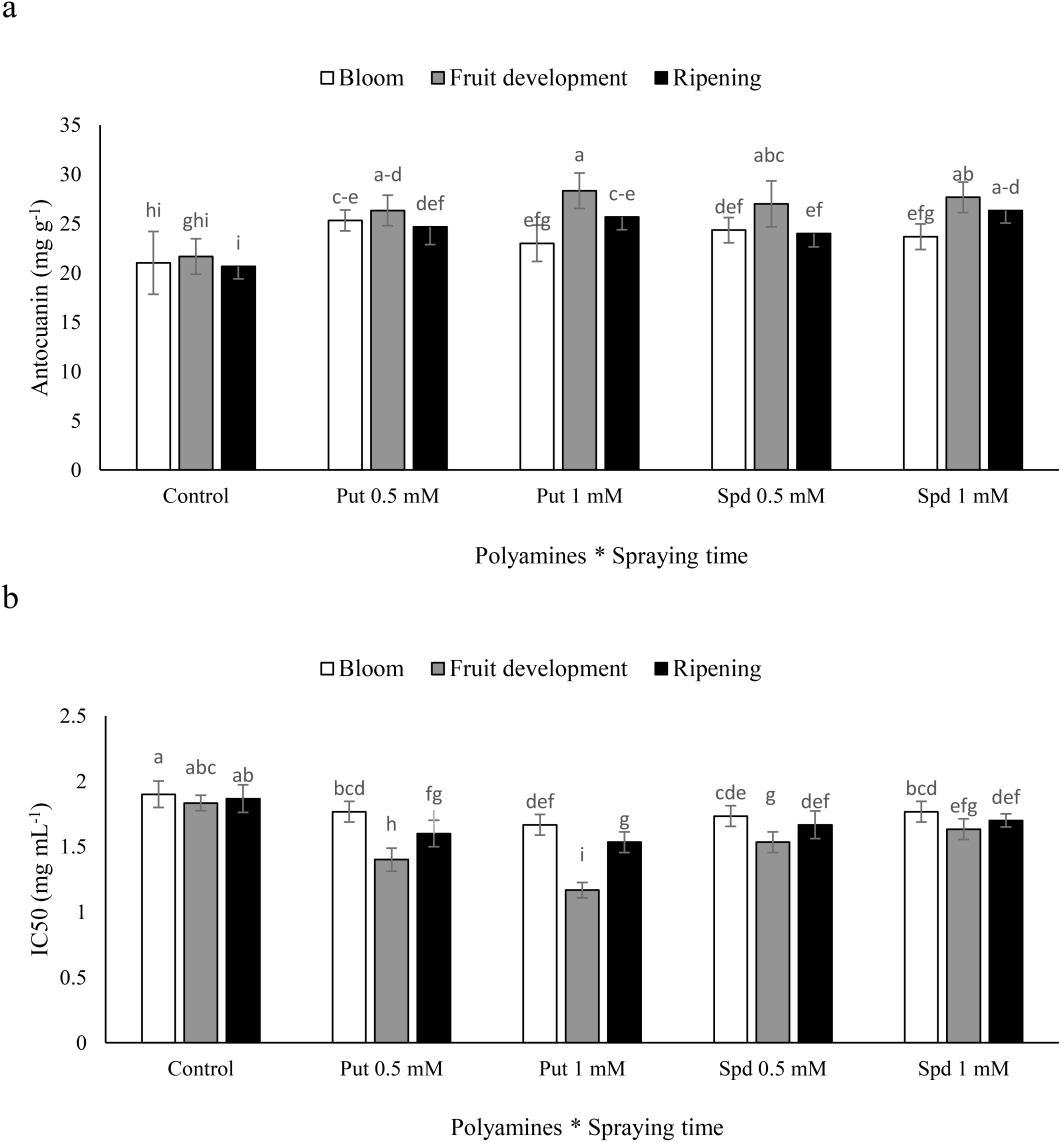
Anthocyanin **(a)** and IC50 **(b)** of blubbery fruits under foliar applied putrescine (Put) and spermidine (Spd). Values are means ± standard error (SE) and different letters show statistically significant differences among treatments at *P ≤* 0.05.

The application of polyamines at different times had varying effects on fruit anthocyanin levels in blueberry plants. The use of Put resulted in a notable increase in anthocyanin content. Specifically, in plants sprayed at the fruit development stage, the application of Put at concentrations of 0.5 and 1 mM, as well as Spd at 0.5 and 1 mM, led to increases of 25%, 31%, 25%, and 28%, respectively, in fruit anthocyanin levels. Furthermore, the application of Put at a concentration of 1 mM was particularly effective, leading to increases of 10%, 25%, and 24% in fruit anthocyanin content at the blooming, fruit development, and harvest stages, respectively, compared to the control (**Figure 3a**). These findings emphasize the importance of considering the timing and concentration of polyamine applications to enhance anthocyanin levels in blueberry fruits. The impact of polyamines sprayed at different times on fruit antioxidant capacity in blueberry plants varied significantly. The use of Put resulted in a notable increase in antioxidant capacity, as evidenced by a decrease in the IC50 value. When plants were sprayed with Put at concentrations of 0.5 and 1 mM, as well as Spd at 0.5 and 1 mM during the fruit development stage, there were decreases of 23%, 36%, 16%, and 10%, respectively, in fruit antioxidant capacity. Moreover, the application of Put at a concentration of 1 mM emerged as a particularly effective treatment, showing diseases of 12%, 36%, and 17% in IC50 at the blooming, fruit development, and harvest stages, respectively, when compared to the control (**Figure 3b**). These results underscore the importance of considering the timing and concentration of polyamine applications in relation to fruit antioxidant capacity in blueberry cultivation.

### Fruit TPC and fruit TFC

**Figure 4:**
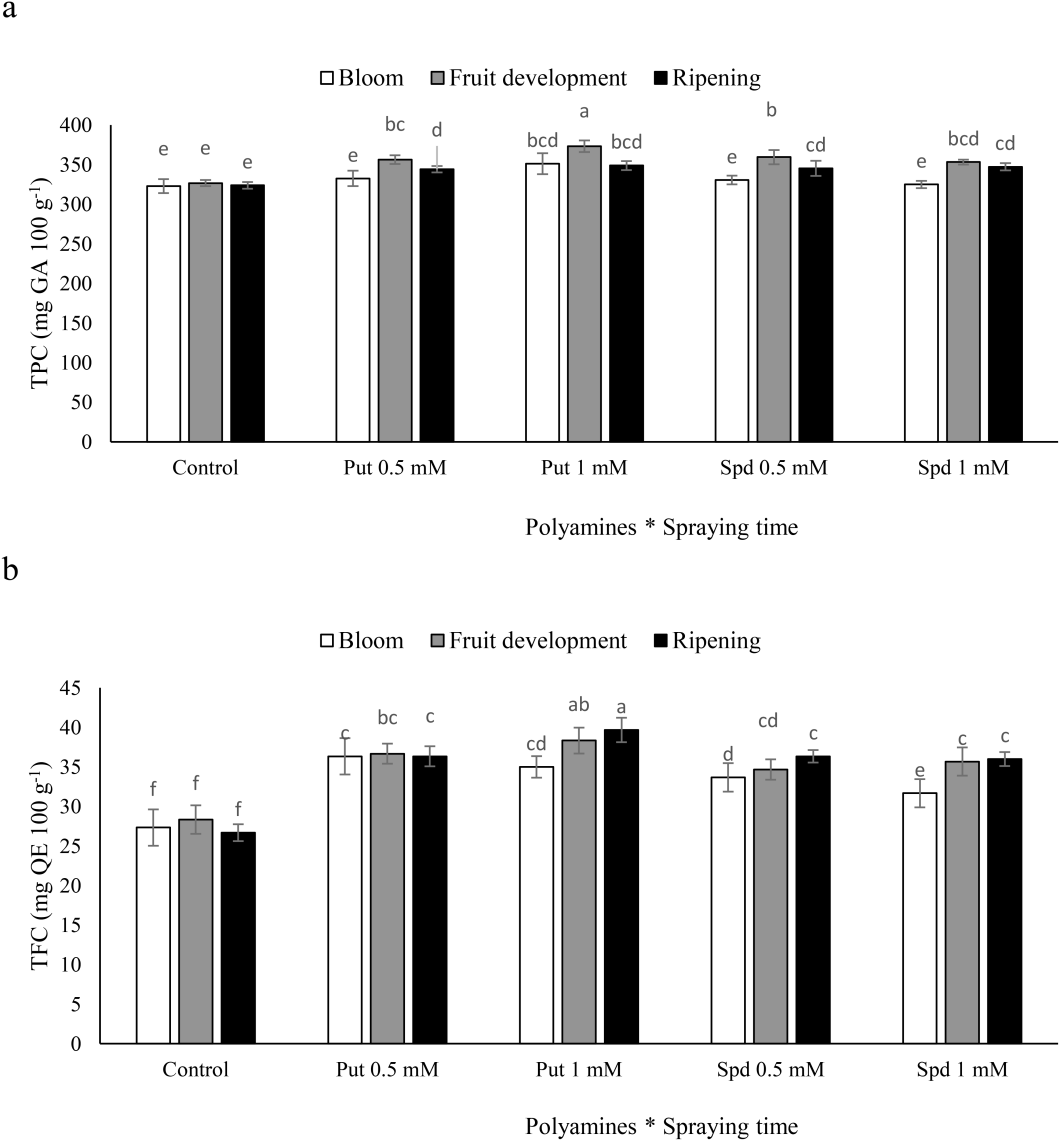
Total phenolic content (TPC, **a**) and total flavonoid content (TFC, **b**) of blubbery fruits under foliar applied putrescine (Put) and spermidine (Spd). Values are means ± standard error (SE) and different letters show statistically significant differences among treatments at *P ≤* 0.05.

The TPC in blueberry plants exhibited varied responses to the timing of polyamine spraying. The highest TPC levels were observed in plants that were sprayed with Put at a concentration of 1 mM during the fruit development stage, resulting in a 15% increase relative to the control (**Figure 4a**). Similarly, the TFC also showed significant increases in response to polyamine treatments. TFC levels increased by 29%, 35%, 22%, and 26% in response to the administration of Put at concentrations of 0.5 and 1 mM and Spd at concentrations of 0.5 and 1 mM, respectively (**Figure 4b**). These findings highlight the potential of polyamines to enhance the phenolic and flavonoid profiles of blueberry plants, particularly when applied at specific concentrations during key growth stages.

### Density and size of leaf stomata

**Figure 5:**
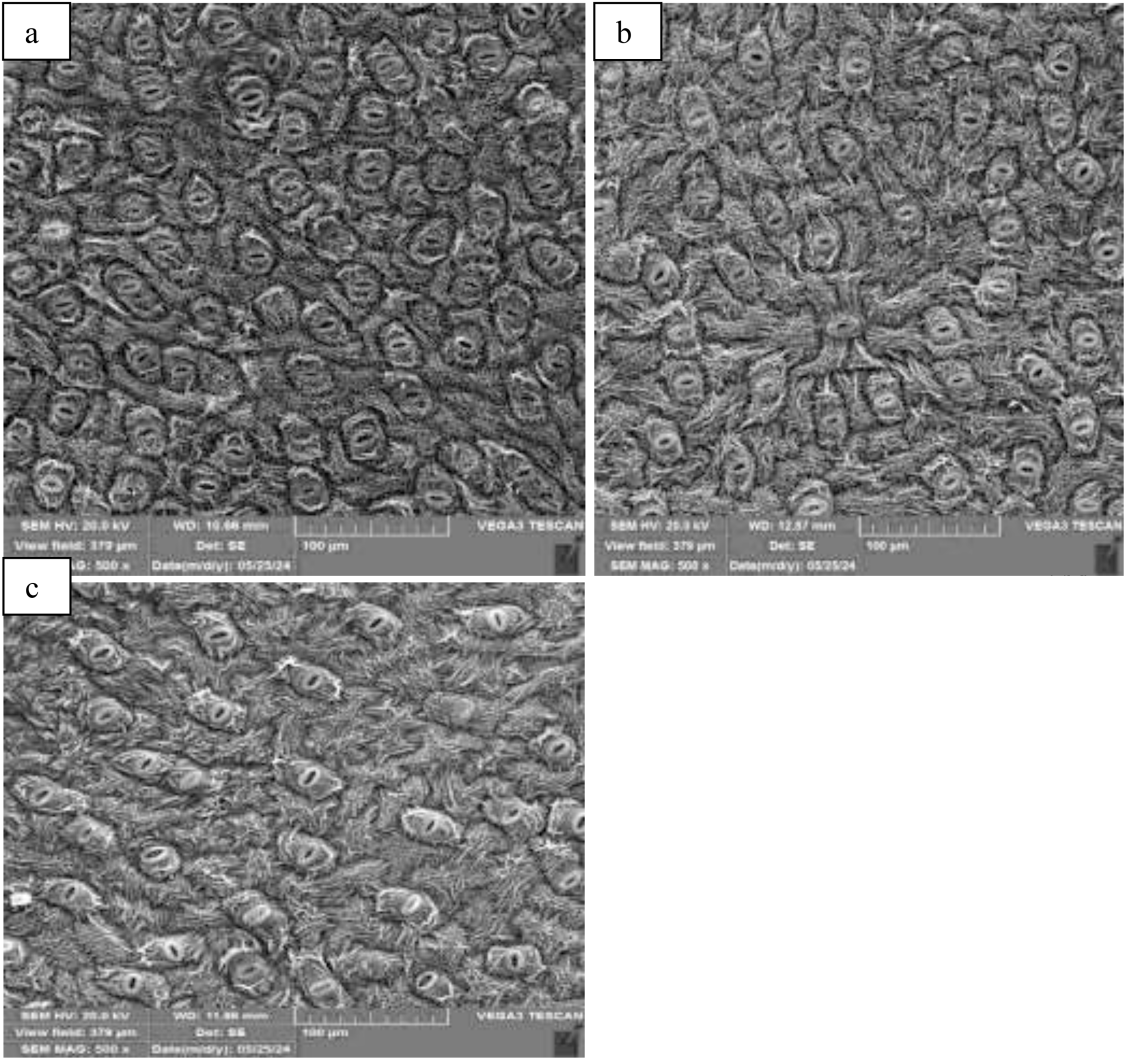
Leaf stomatal distribution under at control **(a)**, spermidine 1 mM **(b)** ad putrescin 1mM **(c)** at fruit development stage.

The application of polyamines had differing effects on stomatal characteristics in blueberry plants. There was a decrease in stomatal density following polyamine spraying, particularly notable at the fruit development stage. For example, when Put was applied at 1 mM, there were declines of 9%, 19%, and 10% in stomatal density at the bloom, fruit development, and harvest stages, respectively (**Figure 5**). Conversely, polyamine treatments led to an increase in stomatal size. The maximum stomatal length of 14 µm was recorded during the fruit development stage when Put was applied at 1 mM, representing a 45% increase compared to the control. Similarly, there was a 40% increase in stomatal width at the same stage and treatment. These enhancements in stomatal size were more pronounced during the fruit development stage compared to other stages (**Table 1**). These results suggest that polyamine applications can influence stomatal characteristics in blueberry plants, with potential implications for plant physiology and water regulation.

**Table 1:** Stomatal properties and leaf minerals of blubbery plants under foliar applied putrescine 732 (Put) and spermidine (Spd).

Polyamine	Time of spraying	Stomatal density	Stomatal length	Stomatal width	Nitrogen	Phosphorous	Potassium
Control	Bloom	283.7 ± 13.5a	11.3 ± 0.78ef	4.67 ± 0.29e	21 ± 1.35h	5.2 ± 0.36e	28.33 ± 1.47g
	Fruit development	255 ± 7.7bcd	13.3 ± 0.59bcd	5.33 ± 0.29cde	25 ± 0.51fg	5.97 ± 0.24cd	32.33 ± 1.56b-e
	Ripening	256.3 ± 6.9bc	14.3 ± 0.59b	5.67 ± 0.59b-e	26.67 ± 1.28cde	6.3 ± 0.33abc	33.67 ± 1.64abc
Put 0.5 mM	Bloom	263.7 ± 7.2b	12.3 ± 0.78de	5.33 ± 0.78cde	24.33 ± 0.78g	5.83 ± 0.3d	30.67 ± 1.06ef
	Fruit development	258.7 ± 9.5bc	13 ± 0.88cd	5 ± 0.88de	25.67 ± 1.28efg	6.23 ± 0.38bcd	31.33 ± 2.12de
	Ripening	279.7 ± 9.2a	11 ± 0.88f	5 ± 0.51de	21.67 ± 1.28h	5.37 ± 0.31e	29 ± 1.35fg
Put 1 mM	Bloom	244.3 ± 9.3def	14 ± 1.02bc	6.67 ± 0.78ab	27.33 ± 1.06bcd	6.37 ± 0.23abc	33 ± 1.53a-d
	Fruit development	224.3 ± 9.4g	16 ± 1.02a	7 ± 0.88a	31 ± 1.84a	6.5 ± 0.41ab	34.33 ± 1.06ab
	Ripening	234.7 ± 10.7f	14 ± 0.88bc	5.67 ± 0.59b-e	28.67 ± 1.79b	6.4 ± 0.23abc	32.67 ± 1.28a-e
Spd 0.5 mM	Bloom	241.7 ± 13.3ef	14.3 ± 1.06b	6.33 ± 0.59abc	27.33 ± 1.28bcd	6.7 ± 0.22a	31.67 ± 0.78cde
	Fruit development	276 ± 6.1a	11.7 ± 0.59ef	4.67 ± 0.59e	22 ± 1.02h	5.3 ± 0.28e	28 ± 1.02g
	Ripening	257.3 ± 8.6bc	13.3 ± 0.59bcd	5.33 ± 0.29cde	26.33 ± 1.28c-f	6.27 ± 0.46abc	32.67 ± 1.28a-e
Spd 1 mM	Bloom	251.7 ± 7.9cde	14.3 ± 0.59b	6 ± 0.51a-d	27.67 ± 1.64bc	6.2 ± 0.18bcd	34.67 ± 0.78a
	Fruit development	256.3 ± 7.9bc	13 ± 0.88cd	5.33 ± 0.29cde	25.33 ± 0.78efg	6.37 ± 0.23abc	33.67 ± 2.06abc
	Ripening	252.7 ± 7.2bcd	14 ± 0.51bc	5.67 ± 0.29b-e	26 ± 1.02def	6.53 ± 0.21ab	33 ± 1.84a-d

Values are means ± standard error (SE) and different letters show statistically significant differences among treatments at *P ≤* 0.05.

### Leaf NPK content

The study’s findings suggest that foliar application of Put and Spd at specific concentrations can significantly impact the nutrient content of plants, particularly in terms of NPK. Among the treatments tested, it was observed that the application of Put at 1 mM resulted in the highest increase in N content, showing a substantial 43% improvement compared to the control. When it comes to phosphorus content, both Put and Spd exhibited positive effects, with Spd slightly outperforming Put. During the fruit development stage, foliar application of Spd at 1 mM led to a notable 25% increase in leaf P content, while Put at the same concentration resulted in a 21% boost. Furthermore, when administered at 0.5 and 1 mM, respectively, Put and Spd both showed favorable effects on leaf K levels. In particular, the treatments increased Put by 16% and 23% and Spd by 20% and 18%, respectively (**Table 1**). Overall, these findings suggest that foliar application of Put and Spd at optimized concentrations could be a viable strategy for improving nutrient uptake and enhancing plant growth.

### PCA

**Figure 6:**
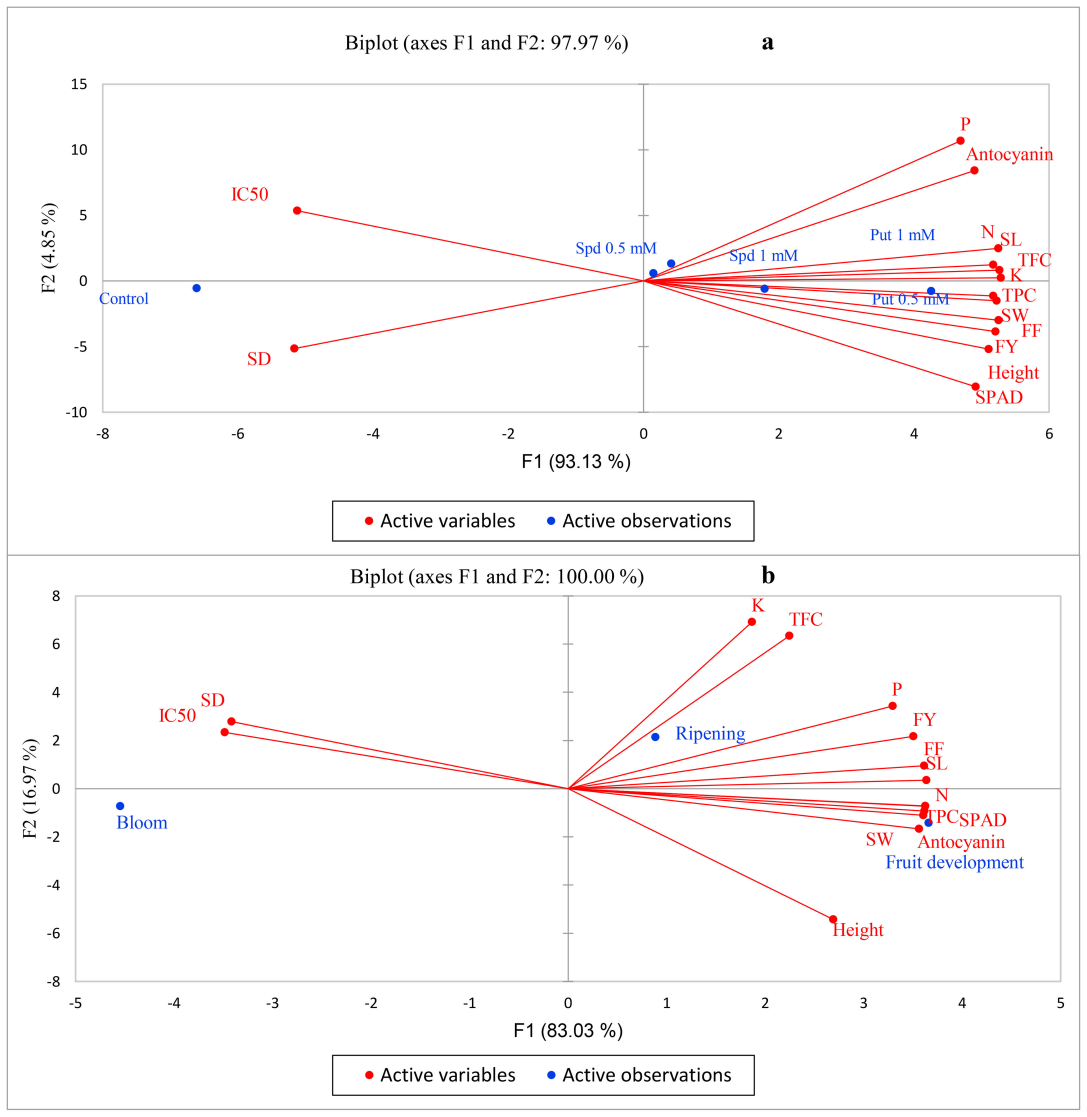
Principal component analysis (PCA) under polyamine levels **(a)** and time of spraying **(b)**. FY, fruit yield; FF, fruit firmness; TPC, total phenolic content; TFC, total flavonoid content; SD, stomatal density; SL, stomatal length; SW, stomatal width; N, nitrogen; P, phosphorous; K, potassium.

The results of the PCA for polyamines indicated that the first component (F1) and the second component (F2) collectively explained 97.9% of the variations observed. Among these components, F1 accounted for a significant 93.1% of the changes, suggesting it played a dominant role in influencing the traits analyzed. Additionally, all traits examined were predominantly explained by F1 and F2, underscoring their importance in characterizing the effects of Put and Spd treatments. It was also noted that all levels of Put and Spd, except for the 1 mM concentration of Spd, were specifically delineated by F1, while F2 justified Spd at 1 mM (**Figure 6a**).

In the PCA analysis of the time of spraying, it was revealed that F1 and F2 accounted for 100% of the variation, with F1 contributing to 83% of the observed changes and F2 explaining approximately 17%. Most traits, with the exception of TFC and leaf K, were attributed to F1, highlighting its predominant role in characterizing the time of spraying effects. Notably, TFC and leaf K were specifically justified by F2, indicating a unique influence of this component on these traits. Furthermore, the bloom and fruit development times were primarily explained by F1, while the harvest time was primarily associated with F2. Additionally, it was observed that IC50 and stomatal density showed negative correlations with other traits, suggesting potential contrasting dynamics in their relationships within the dataset (**Figure 6b**).

### Heat map

**Figure 7:**
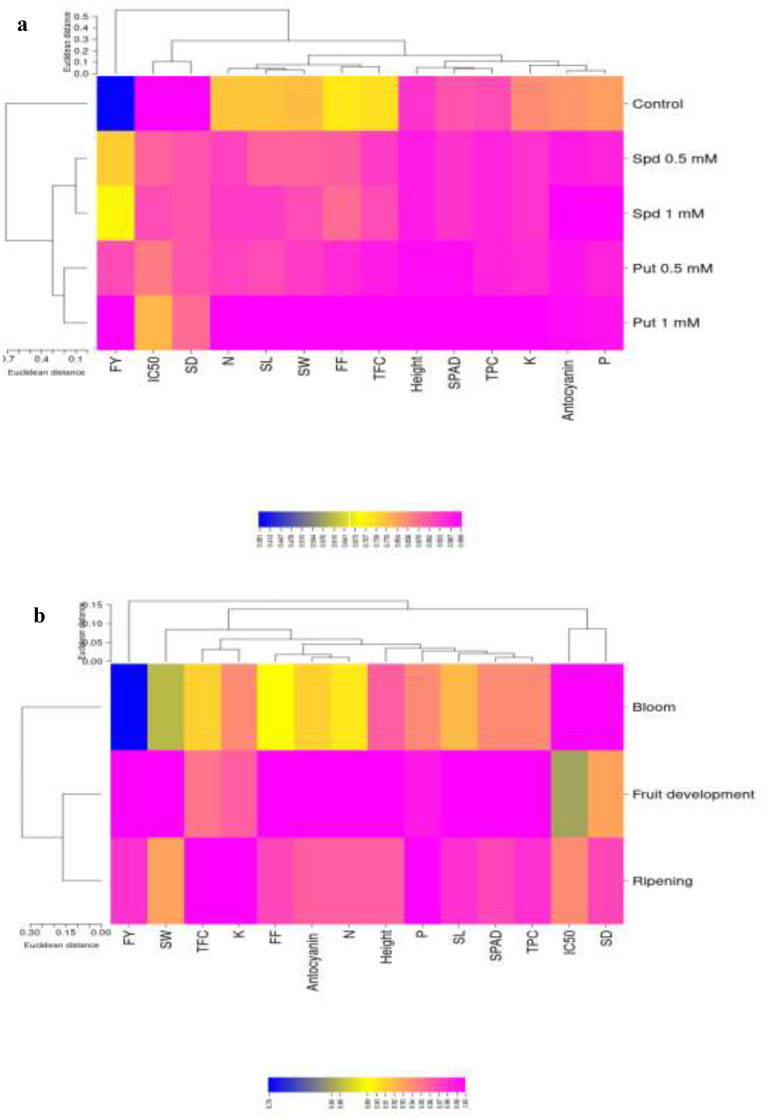
Heat map analysis under polyamine levels **(a)** and time of spraying **(b)**. FY, fruit yield; FF, fruit firmness; TPC, total phenolic content; TFC, total flavonoid content; SD, stomatal density; SL, stomatal length; SW, stomatal width; N, nitrogen; P, phosphorous; K, potassium.

The results of the heat map analysis revealed that fruit yield exhibited the highest variability under the different treatments, indicating its sensitivity to the experimental conditions. Following fruit yield, fruit firmness and TFC showed the highest variability in response to polyamine treatments. However, when considering fruit yield as the most sensitive trait, it was observed that fruit firmness and leaf K displayed the maximum variability (**Figure 7a**). Conversely, plant height exhibited the least variability among the traits analyzed.

In terms of clustering analysis, it was observed that Put at concentrations of 0.5 and 1 mM formed one cluster, while Spd at 0.5 and 1 mM constituted a separate cluster. These two clusters were found to be significantly different from the control, indicating distinct effects of the different polyamine treatments on the traits under investigation. This clustering pattern suggests that the responses of the plants to Put and Spd treatments at varying concentrations were distinct and led to significant differences in the measured attributes. Overall, the heat map and clustering analyses provide valuable insights into the impact of polyamine treatments on various plant traits, with fruit yield, fruit firmness, and leaf K emerging as key factors demonstrating significant variability under the experimental conditions (**Figure 7b**). Understanding the relationships between these traits and the effects of different polyamines can aid in optimizing plant growth and productivity through targeted treatments and interventions.

## Discussion

Polyamines like Put and Spd can enhance plant height by regulating cell division and elongation. They also enhance protein synthesis, allowing for structural protein production for cell wall expansion. Polyamines interact with plant hormones, influencing hormone signaling pathways that promote cell elongation and plant height (Tyagi et al., 2023). They help plants cope with environmental stresses, allowing them to allocate more resources towards growth. They also enhance nutrient uptake and assimilation, providing essential building blocks for cell growth (Jangra et al., 2023). Liu et al. (2023) demonstrated the beneficial effect of Spd on cherry tomato plant height, which supports our findings. Likely due to differences in cell division, interaction with signaling pathways, metabolic processing, stress tolerance, and the concentration and method of application (Vaka et al., 2020). Strategic timing of foliar application during fruit development optimizes growth and height, leveraging the plant’s physiological processes (Wang et al., 2020).

Foliar spraying of Spd and Put increased the chlorophyll index in blubbery plants by activating enzymes involved in chlorophyll biosynthesis. These polyamines protect chlorophyll molecules from deterioration, preserving their structural integrity (Islam et al., 2020). They also improve photosynthetic efficiency by promoting electron transport, ATP production, and carbon absorption. Polyamines also enhance the intake of essential elements like nitrogen, magnesium, and iron, enhancing plant development, growth, and photosynthetic efficiency (Yousefi et al., 2019). Put and Spd can improve the development, growth, and photosynthetic efficiency of plants by modifying biochemical processes associated with the metabolism of chlorophyll and photosynthesis (Jing et al., 2020; Balci et al., 2023). Khodabakhshi et al. (2023) showed that Put significantly increased Chl content in *Indigofera tinctoria*, which is consistent with the findings of the present study. Timing the application of polyamines correctly plays a vital role in improving photosynthetic activity. This research focuses on the optimal time frame for polyamines to achieve maximum photosynthesis rates in fruit development. During the early to mid-stage of growth, polyamines can enhance chlorophyll biosynthesis, increase fruit content, and improve photosynthetic efficiency (Nandy et al., 2022). During bloom time, they support flower development and fruit set, but their impact on chlorophyll concentration and photosynthesis may be less pronounced. Harvest time may have limited benefits, as mature plants may not respond as well (Fortes and Agudelo-Romero, 2018). Similarly, Mahdavian et al. (2021) showed that exogenous application of Put positively enhances a photosynthesis rate in two citrus rootstocks.

Foliar-applied Put and Spd enhanced fruit yield. These polyamines are essential plant growth regulators that regulate cell division, differentiation, and stress responses. They can positively impact fruit yield through cell division and elongation, flower and fruit development, nutrient uptake and assimilation, photosynthesis and carbohydrate metabolism, and stress tolerance (Huang et al., 2024). By enhancing these processes, polyamines stimulate fruit growth, increase fruit size and yield, improve nutrient availability, and enhance photosynthesis and carbohydrate metabolism. They also act as antioxidants, helping plants cope with environmental stressors and ensuring healthy growth and fruit production. Overall, foliarly applied polyamines contribute to increased fruit yield and overall plant productivity (Piñero et al., 2021).

Polyamines like Put and Spd have been found to increase the firmness of blueberries by improving cell wall structure and integrity. They can crosslink with cell wall components like pectins and cellulose, leading to increased cell wall strength and firmness (Hadjipieri et al., 2021). Polyamines also improve membrane stability and protect cells from oxidative damage, contributing to fruit firmness (Singh et al., 2022). They regulate the expression of genes related to cell wall metabolism and fruit ripening processes, promoting the synthesis of cell wall components and inhibiting enzymes that degrade cell wall materials. Put has a stronger regulatory impact than Spd and a smaller molecular weight, making it more effective than Spd (González-Hernández et al., 2022). This suggests that polyamines can be used in the horticulture sector to enhance fruit quality and shelf life. The optimal timing of polyamine application can improve fruit quality, meet market needs, and increase overall competitiveness in the horticulture business (Hanif et al., 2020; Manjunatha et al., 2024).

Foliar-applied Put and Spd led to increased anthocyanin and antioxidant capacity. These polyamines stimulate the biosynthesis of anthocyanins, enhancing their nutritional value and antioxidant capacity. Put and Spd possess antioxidant properties that scavenge free radicals and reduce oxidative stress in cells, helping combat oxidative stress associated with chronic diseases and aging (Mishra et al., 2022; Zeynali et al., 2023). These properties also contribute to the nutritional value of blueberries, making them appealing to consumers seeking health-promoting products and meet the growing demand for antioxidant-rich food options (Prajapati et al., 2024). Similarly, increased antioxidant potential has been reported on guava fruit (Thapa et al., 2023). The optimal time to spray Put and Spd during fruit development is during fruit maturity, as it is connected to vigorous growth and metabolic activities. Growers can optimize the effects of these polyamines to improve fruit quality, maximize nutrient absorption, and foster stress tolerance by focusing on this critical developmental period (Tavallali et al., 2024).

Put and spermidine increased the phenol and flavonoid content of blueberries through various biochemical pathways. These pathways include the regulation of the phenylpropanoid pathway, activating antioxidant enzymes, modulating gene expression, and stimulating the production of phenols and flavonoids (Kołton et al., 2022; Nandy et al., 2022). They also interact with polyamine metabolism, enhancing the nutritional and antioxidant properties of blueberries (Zeynali et al., 2023). Understanding these mechanisms can optimize their application for enhancing blueberry nutritional value and health benefits (Nandy et al., 2022). Consistent with the findings of this study, Mishra et al. (2022) shown that exogenous Put and Spd treatment enhanced phenolic compounds. Optimizing the timing of polyamine application during blueberry development is essential for maximizing their uptake and effectiveness, significantly increasing the accumulation of phenolic compounds in the fruit, improving its phytochemical profile and health-promoting properties (Lobos et al., 2021).

Polyamines, such as putrescine and spermidine, can decrease stomatal density and increase stomatal size in plants due to their role in cell division regulation, inhibition of stomatal development, and promotion of cell expansion (Yang et al., 2022). They also affect cell wall properties, leading to increased stomatal size and enlargement of individual cells. Changes in stomatal density can affect leaf surface stomatal spacing and the role of guard cells (Torabian et al., 2018; Xu-Dong et al., 2023). Previously, leaf nutrition by selenium has been reported to decreased stomatal density and increased stomatal size (Nasirzadeh et al., 2022). Polyamines could potentially improve plant resilience, resource efficiency, and productivity in agricultural systems by manipulating stomatal characteristics. Further research is needed to fully understand their mechanisms and potential benefits (Yang et al., 2022).

Polyamines, put and Spd, play a crucial role in nutrient uptake and utilization in plants. They regulate nutrient transporters in roots, stimulate root growth, and influence enzyme activity, facilitating the conversion and utilization of nutrients within plant cells (Zhong et al., 2023). Put and Spd are implicated in stress responses, helping plants cope with nutrient stress conditions by enhancing nutrient uptake efficiency (Rakbar et al., 2024). Put was more effective for leaf nitrogen uptake than Spd, stimulating chlorophyll biosynthesis and protein synthesis, leading to higher nitrogen levels. Put also enhances nitrogen use efficiency, optimizing resource allocation, leading to higher levels and improved plant growth (Hussein et al., 2023; Rakbar et al., 2024). Similarly, Sharma et al. (2023) observed that Put had a greater effect on enhancing N uptake than Spd. Foliar application of nutrients, especially during fruit development, supports optimal fruit growth, development, quality, and yield, enhancing crop productivity and overall agricultural success (Bons and Sharma, 2023).

## Conclusions

This study reveals the potential of foliar application of putrescine and spermidine at key growth stages for blueberry growers. The research emphasizes the importance of timing when applying polyamines like putrescine and spermidine, indicating that the fruit development stage is the most favorable for foliar spraying. This information can help growers plan and execute fertilization strategies during critical growth stages. Putrescine, particularly, has been found to improve blueberry plant growth and fruit yield, aiding growers in making informed decisions when selecting fertilization options. The study also emphasizes the need for a holistic approach to crop management, considering factors like stomatal characteristics, anthocyanin content, and overall fruit quality. By demonstrating the multifaceted benefits of strategic foliar application, the study encourages producers to adopt more comprehensive strategies that encompass various aspects of plant health and productivity. The insights from this study have the potential to revolutionize blueberry cultivation practices, offering a pathway towards increased yield and improved fruit quality. By incorporating the findings into farming techniques, growers can harness the benefits of polyamine application at key growth stages to optimize blueberry production and meet the demands of an ever-evolving agricultural market.

## Data Availability

The original contributions presented in the study are included in the article/supplementary material. Further inquiries can be directed to the corresponding author.
